# Association Between Large Hernia Size and Severe Postoperative Complications After Type III/IV Hiatal Hernia Repair

**DOI:** 10.7759/cureus.96733

**Published:** 2025-11-13

**Authors:** Seitaro Nishimura, Shinya Asami, Tetsuya Kagawa, Kazuki Hara, Rentaro Doi, Toru Noso, Norihisa Takakura

**Affiliations:** 1 Department of Surgery, Fukuyama City Hospital, Fukuyama, JPN

**Keywords:** esophageal hiatal hernia, esophageal perforation, large hernia, postoperative complication, postoperative recurrence, reoperation

## Abstract

Introduction

Large Type III and IV esophageal hiatal hernias often require surgical repair because of organ herniation and related symptoms. Although the procedure is benign, it is associated with a significant risk of complications, particularly in large hernias. This study aimed to clarify risk factors for severe postoperative complications in patients requiring reoperation following surgical repair of Type III and IV esophageal hiatal hernias.

Methods

We retrospectively reviewed 34 patients who underwent repair of Type III (n = 21) or IV (n = 13) esophageal hiatal hernias (2014-2024). Clinical data and preoperative computed tomography (CT) measurements of the hiatal length and hernial sac area were analyzed. Operative time was dichotomized at the cohort median, and variables with p < 0.10 in univariable tests were entered into a Firth’s penalized logistic regression to identify predictors of prolonged operative time.

Results

Severe postoperative complications (Clavien-Dindo Grade IIIb) occurred in three patients, all with Type IV hernias and larger hiatus lengths and hernia sac areas. Hernia sac area correlated positively with operative time (ρ = 0.60, p < 0.001). In multivariable analysis using Firth’s penalized logistic regression, hernia sac area was independently associated with prolonged operative time (OR 15.9, p < 0.001), consistent with univariable findings.

Conclusion

A large hernia size was associated with prolonged operative time and increased risk of severe postoperative complications. Preoperative assessment of hernia size may facilitate surgical planning and improve outcomes.

## Introduction

The incidence of esophageal hiatal hernia has increased in recent years, partly because of population aging and rising prevalence of obesity [1].

Type I sliding hiatal hernia is often associated with severe gastroesophageal reflux disease (GERD). However, medical therapies, including the use of potassium-competitive acid blockers, such as vonoprazan, have been shown to effectively manage GERD symptoms and prevent disease progression [2,3]. In contrast, Type III and IV hiatal hernias are characterized by herniation of a large volume of stomach and other abdominal organs into the thorax. This produces mechanical compression of the lungs and mediastinum, distortion of the gastroesophageal junction with severe reflux and aspiration risk, and a higher likelihood of gastric outlet obstruction or volvulus resulting in ischemia/strangulation [4]. In cases of large hernias, an "upside-down stomach" may occur, and patients may experience symptoms beyond GERD, including decreased quality of life and anemia, often necessitating surgical intervention [5-7].

Although considered elective, hiatal hernia repair, especially in large hernias, carries substantial perioperative risk [8]. Furthermore, owing to the difficulty in securing an adequate surgical field, the need for mediastinal dissection, and the technical demands of fundoplication, hiatal hernia repair can result in a wide range of complications, including bleeding, perforation, and recurrence [9]. In some cases, reoperation may be required for these complications. Reported rates of major postoperative complications after repair of giant (Type III/IV) paraesophageal hernias vary widely across eras and centers, ranging from approximately 1%-12% in contemporary high-volume series to approximately 6%-19% in earlier cohorts; by contrast, emergency repairs carry substantially higher risks, with major morbidity of 8%-30% and mortality up to 8%-22% [10-12].

Despite these challenges, few studies have specifically investigated the risk factors for severe postoperative complications following hiatal hernia repair. Thus, this study aimed to assess the incidence of postoperative complications and identify any associated risk factors in patients who underwent surgical repair for Type III and IV hiatal hernias at our institution.

## Materials and methods

Participants and data collection

Hiatal hernias were classified according to the conventional anatomic classification (Types I-IV), based on the position of the gastroesophageal junction and the extent of herniation through the diaphragmatic hiatus [13] (Table 1).

**Table 1:** Classification of hiatal hernias (Types I–IV) GEJ: gastroesophageal junction.

Type	Description
I	The GEJ and a portion of the stomach slide upward through the esophageal hiatus into the thoracic cavity.
II	The gastric fundus herniates alongside the esophagus, while the GEJ remains in its normal intra-abdominal position.
III	Both the GEJ and a portion of the stomach herniate through the hiatus, features of both Types I and II.
IV	In addition to the stomach, other abdominal organs also herniate into the thoracic cavity.

**Figure 1:**
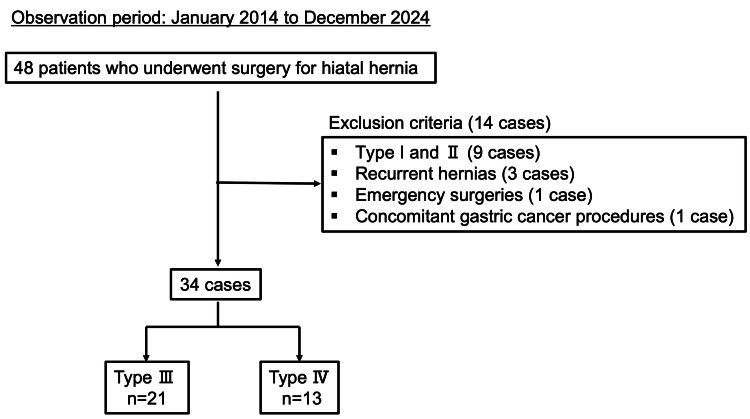
Flowchart of patient selection for the analysis of Type III and IV hiatal hernia surgery

This retrospective study included 34 patients who underwent surgical repair of Type III (n = 21) or IV (n = 13) hiatal hernias at our institution between January 2014 and December 2024. Among the 48 patients who underwent surgery for hiatal hernia during this period, 14 were excluded based on the following criteria: Type I or II hernia (n = 9), recurrent hernia (n = 3), need for emergency surgery (n = 1), and concomitant gastric cancer (n = 1) (Figure 1).

Almost all procedures were performed using a laparoscopic approach by experienced surgeons in our upper gastrointestinal team. The fundamental surgical technique remained consistent throughout the 10-year study period. However, some procedural details evolved over time: mesh reinforcement was used more frequently in earlier cases but has been discontinued in recent years, and the preferred fundoplication method shifted from anterior to Toupet in accordance with current practice. Perioperative management protocols remained unchanged during the entire study period. Preoperative esophagogastroduodenoscopy was routinely performed to evaluate the presence of reflux esophagitis, Barrett’s epithelium, or concomitant gastric pathology, and to confirm the anatomical features of the hernia.

Data collected from medical records included patient age, sex, body mass index (BMI), American Society of Anesthesiologists Physical Status (ASA-PS), comorbidities (hypertension, dyslipidemia, diabetes mellitus, COPD/asthma, cardiovascular disease, chronic kidney disease, cerebrovascular disease, and malignancy), surgical approach, operative time, length of hospital stay, presence of postoperative complications, and hernia recurrence. Additionally, preoperative coronal-plane computed tomography (CT) was used to measure the hiatus length (maximum diameter of the esophageal hiatus) and hernia sac area (maximum cross-sectional area of the hernia sac), which were assessed by one gastrointestinal surgeon blinded to postoperative outcomes.

Statistical analysis

Continuous variables were expressed as median (interquartile range) and compared using the Mann-Whitney U test. Categorical variables were analyzed using Fisher’s exact test, particularly when appropriate for small sample sizes or when the expected cell count was <5. Operative time was dichotomized at the cohort median. Covariates with p < 0.10 in univariable testing were entered into a multivariable model; to mitigate small-sample bias and potential separation, we used Firth’s penalized logistic regression to identify independent predictors of prolonged operative time. Statistical significance was set at a two-tailed p-value of <0.05. All analyses were performed using EZR v.1.56, which is a graphical user interface for R.

## Results

Comparison of Type III and Type IV hiatal hernias

Of the 48 patients who underwent surgical repair for esophageal hiatal hernias at our institution between January 2014 and December 2024, 34 were selected for evaluation after applying the exclusion criteria and were classified into the Type III and Type IV groups for comparison (Table 2).

**Table 2:** Comparison of patient characteristics, surgical approaches, and outcomes between Type III and IV hiatal hernias ^*^Mann-Whitney U test. ^†^Fisher's exact test. BMI: body mass index, ASA-PS: American Society of Anesthesiologists Physical Status, CD: Clavien-Dindo classification.

Variable	Type Ⅲ (n = 21)	Type Ⅳ (n = 13)	p-value
Age (median (IQR))	77.00 (73.00, 83.00)	78.00 (76.00, 82.00)	0.657^*^
Sex: female (%)	21 (100.0)	9 (69.2)	0.015^†^
Sex: male (%)	0 (0.0)	4 (30.8)	
BMI (median (IQR))	23.03 (20.82, 25.50)	22.75 (21.88, 23.62)	0.684^*^
ASA-PS: 1 (%)	1 (4.8)	0 (0.0)	0.269^†^
ASA-PS: 2 (%)	14 (66.7)	12 (92.3)	
ASA-PS: 3 (%)	6 (28.6)	1 (7.7)	
Comorbidity: yes (%)	15 (71.4)	12 (92.3)	0.21^†^
Comorbidity: no (%)	6 (28.6)	1 (7.7)	
Hiatus length (cm) (median (IQR))	3.60 (2.80, 4.00)	4.90 (4.37, 6.40)	0.001^*^
Hernia sac area (cm^2^) (median (IQR))	61.30 (36.55, 71.90)	127.06 (92.35, 153.93)	<0.001^*^
Lap (%)	20 (95.2)	11 (84.6)	0.474^†^
Lap → open (%)	1 (4.8)	1 (7.7)	
Open (%)	0 (0.0)	1 (7.7)	
Fundoplication: anterior (%)	12 (57.1)	5 (38.5)	0.281^†^
Fundoplication: Toupet (%)	9 (42.9)	7 (53.8)	
Fundoplication: no (%)	0 (0.0)	1 (7.7)	
Mesh: yes (%)	5 (23.8)	6 (46.2)	0.262^†^
Mesh: no (%)	16 (76.2)	7 (53.8)	
Operative time (median (IQR))	170.00 (145.00, 222.00)	206.00 (157.00, 227.00)	0.107^*^
Length of stay (day) (median (IQR))	9.00 (7.00, 11.00)	9.00 (7.00, 12.00)	0.506^*^
CD grade: 0 (%)	20 (95.2)	9 (69.2)	0.026^†^
CD grade: I (%)	0 (0.0)	1 (7.7)	
CD grade: Ⅱ (%)	1 (4.8)	0 (0.0)	
CD grade: Ⅲb (%)	0 (0.0)	3 (23.1)	
Recurrence: yes (%)	1 (5.3)	3 (23.1)	0.279^†^
Recurrence: no (%)	18 (94.7)	10 (76.9)	

Although the majority of patients were female, a small number of male patients were observed in the Type IV group. No significant differences were observed between the Type III and Type IV groups in terms of age, BMI, ASA-PS score, or comorbidities. The maximal hiatal diameter and hernia sac area were measured using coronal CT images, and both parameters were significantly larger in the Type IV group than in the Type III group. Laparoscopic surgery was performed in nearly all cases. Although the choice of the fundoplication method (Toupet vs. anterior) and the use of mesh varied depending on the time period, no significant differences were observed between the two groups. Regarding surgical outcomes, no significant differences were observed in operative time or length of hospital stay. Postoperative complications were assessed using the Clavien-Dindo classification, and three patients in the Type IV group experienced Grade IIIb complications requiring reoperation. Recurrence was observed in four patients: three patients who required reoperation and one additional patient who developed late recurrence.

Analysis of Grade IIIb complications and risk factors

To explore common factors among those patients who required reoperation, we focused on three patients who developed Grade IIIb complications. The clinical characteristics of these three patients are summarized in Table 3.

**Table 3:** Clinical characteristics and reoperation details of patients with Clavien-Dindo Grade IIIb complications POD: postoperative day; Reop: reoperation; F: female

Case	Age, sex	Fundoplication	Mesh	Intraoperative findings	Complication	POD (onset/reop)	Reoperation procedure
1	81, F	Toupet	No	Adhesions in hernia sac	Recurrence	5/6	Open repair (anterior fundoplication and gastropexy)
2	82, F	Anterior	No	Adhesions in hernia sac	Recurrence	2/6	Laparoscopic to open repair with gastropexy
3	69, F	Anterior	Yes	Adhesions + short esophagus	Esophageal perforation	10/14	Thoracoscopic drainage, hernia closure, jejunostomy

**Figure 2:**
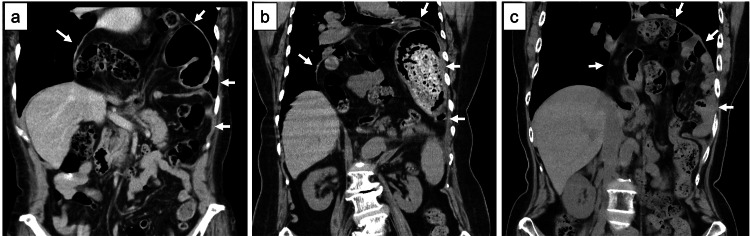
Preoperative computed tomography images of the three representative cases showing large hiatus lengths and hernia sac areas (a) Case 1. (b) Case 2. (c) Case 3. White arrows indicate the margins of the esophageal hiatus and the extent of the hernia sac.

Among the three cases, two underwent anterior fundoplication and one underwent Toupet fundoplication. Mesh reinforcement was used in one case, whereas the other two were repaired without mesh. There were no differences in the type of suture materials used, and intraoperative adhesions were observed in all three cases. All three patients exhibited both large hiatus lengths and large hernial sac areas, as clearly demonstrated on the preoperative CT images (Figure 2).

Patients 1 and 2 required reoperation due to symptomatic early postoperative recurrence, whereas Patient 3 developed an esophageal perforation leading to hernia sac infection and empyema, resulting in a highly complicated clinical course.

To identify the risk factors for major complications, the three Grade IIIb cases were compared with the remaining 31 cases (Grades 0-II) (Table 4).

**Table 4:** Comparison of clinical characteristics, surgical approaches, and outcomes between patients with Clavien-Dindo Grade ≤ II and Grade IIIb complications ^*^Mann-Whitney U test. ^†^Fisher's exact test. CD: Clavien-Dindo classification, BMI: body mass index, ASA-PS: American Society of Anesthesiologists Physical Status.

Variable	CD Grade ≦ Ⅱ (n = 31)	CD Grade Ⅲb (n = 3)	p-value
Age (median (IQR))	77.00 (74.00, 82.50)	81.00 (75.00, 81.50)	0.903^*^
Sex: female (%)	27 (87.1)	3 (100.0)	1^†^
Sex: male (%)	4 (12.9)	0 (0.0)	
BMI (median (IQR))	23.03 (20.59, 24.65)	22.75 (22.48, 24.85)	0.564^*^
ASA-PS: 1 (%)	1 (3.2)	0 (0.0)	1^†^
ASA-PS: 2 (%)	23 (74.2)	3 (100.0)	
ASA-PS: 3 (%)	7 ( 22.6)	0 (0.0)	
Comorbidity: yes (%)	24 (77.4)	3 (100.0)	1^†^
Comorbidity: no (%)	7 (22.6)	0 (0.0)	
Type: Ⅲ (%)	21 ( 67.7)	0 ( 0.0)	0.048†
Type: Ⅳ (%)	10 ( 32.3)	3 (100.0)	
Hiatus length (cm) (median (IQR))	3.80 (3.04, 4.70)	6.40 (5.20, 6.95)	0.068^*^
Hernia area (cm^2^) (median (IQR))	67.18 (49.75, 94.12)	170.32 (162.12, 206.34)	0.007^*^
Lap (%)	28 (90.3)	3 (100.0)	1^†^
Lap → open (%)	2 (6.5)	0 (0.0)	
Open (%)	1 (3.2)	0 (0.0)	
Fundoplication: anterior (%)	16 (51.6)	1 (33.3)	0.636^†^
Fundoplication: Toupet (%)	14 (45.2)	2 (66.7)	
Fundoplication: no (%)	1 (3.2)	0 (0.0)	
Mesh: yes (%)	10 (32.3)	1 (33.3)	1^†^
Mesh: no (%)	21 (67.7)	2 (66.7)	
Operative time (min) (median (IQR))	173.00 (146.50, 220.50)	306.00 (259.00, 307.50)	0.027^*^
Length of stay (day) (median (IQR))	9.00 (7.00, 10.00)	31.00 (24.50, 40.00)	0.003^*^

**Figure 3:**
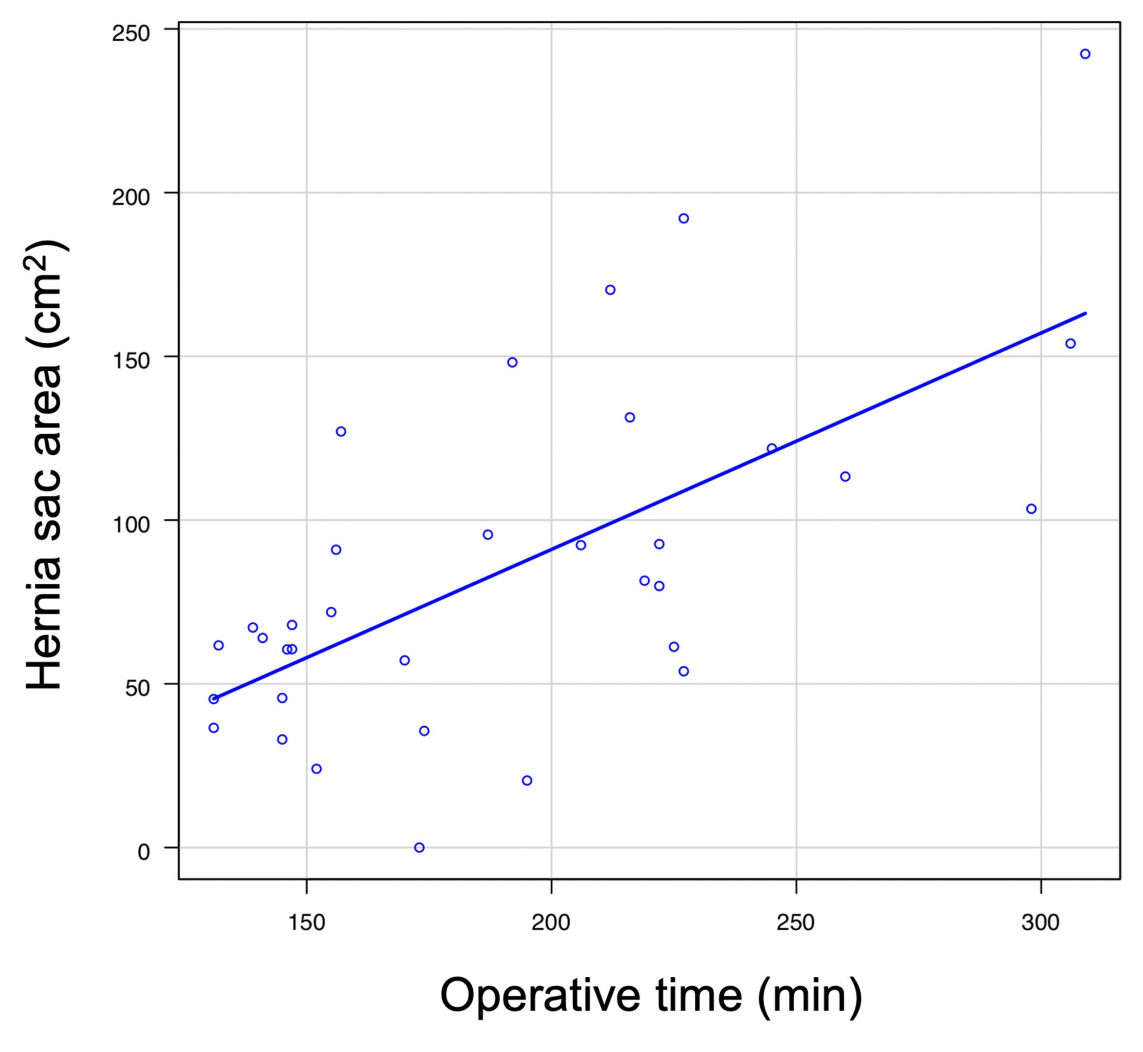
Positive correlation between hernia sac area and operative time

All Grade IIIb cases were classified as Type IV, and significant differences were observed in hernial sac area, operative time, and length of hospital stay. Although the number of reoperation cases was limited (n = 3), we further investigated the relationship between hernial sac size and operative time. A positive correlation was observed between the hernial sac area and operative time (Spearman’s rank correlation coefficient, 0.6; p < 0.001) (Figure 3).

Patients were stratified based on the median operative time, and univariate analysis revealed that the hernial sac area was the only variable that showed a significant association (Table 5).

**Table 5:** Univariate analysis of clinical and surgical factors associated with operative time ^†^Fisher's exact test. BMI: body mass index, ASA-PS: American Society of Anesthesiologists Physical Status, CD: Clavien-Dindo classification.

Variable	Operative time	Operative time (≥median) (n = 17)	p-value
Age < 80 (%)	11 (64.7)	10 (58.8)	1^†^
Age ≥ 80 (%)	6 (35.3)	7 (41.2)	
Sex: female (%)	15 (88.2)	15 (88.2)	1^†^
Sex: male (%)	2 (11.8)	2 (11.8)	
BMI < 22 (%)	8 (47.1)	4 (23.5)	0.282^†^
BMI ≥ 22 (%)	9 (52.9)	13 (76.5)	
ASA-PS: 1-2 (%)	11 (64.7)	16 (94.1)	0.085^†^
ASA-PS: 3 (%)	6 (35.3)	1 (5.9)	
Comorbidity: yes (%)	13 (76.5)	14 (82.4)	1^†^
Comorbidity: no (%)	4 (23.5)	3 (17.6)	
Type: Ⅲ (%)	13 ( 76.5)	8 (47.1)	0.157^†^
Type: Ⅳ (%)	4 ( 23.5)	9 (52.9)	
Hiatus length < median (%)	11 (64.7)	6 (35.3)	0.169^†^
Hiatus length ≥ median (%)	6 (35.3)	11 (64.7)	
Hernia sac area < median (%)	14 (82.4)	3 (17.6)	<0.001^†^
Hernia sac area ≥ median (%)	3 (17.6)	14 (82.4)	
CD grade: 0-II (%)	17 (100.0)	14 (82.4)	0.227^†^
CD grade: IIIb (%)	0 (0.0)	3 (17.6)	
Lap (%)	15 (88.2)	16 (94.1)	1†
Lap → open (%)	1 (5.9)	1 (5.9)	
Open (%)	1 (5.9)	0 (0.0)	
Fundoplication: anterior (%)	6 (35.3)	11 (64.7)	0.169^†^
Fundoplication: Toupet (%)	10 (58.8)	6 (35.3)	
Fundoplication: no (%)	1 (5.9)	0 ( 0.0)	
Mesh: yes (%)	6 (35.3)	5 (29.4)	1^†^
Mesh: no (%)	11 (64.7)	12 (70.6)	
Recurrence: yes (%)	1 (6.7)	3 (17.6)	0.603^†^
Recurrence: no (%)	14 (93.3)	14 (82.4)	

On Firth’s penalized logistic regression, hernia sac area was significantly associated with prolonged operative time (OR, 15.9; 95% CI, 3.2-114.9; p <0.001), whereas ASA-PS was not significant (p = 0.10) (Table 6).

**Table 6:** Multivariate analysis (Firth’s penalized logistic regression) of factors associated with prolonged operative time ASA-PS: American Society of Anesthesiologists Physical Status.

Variable	OR	95% CI	p-value
ASA-PS (1-2 vs 3)	0.16	0.01-1.41	0.102
Hernia sac area (cm^2^) (<median vs ≥median)	15.9	3.2-114.9	<0.001

The results indicate that a larger hernia sac area is an independent risk factor for prolonged operative time, even after bias correction for the small sample size.

## Discussion

In this study, although based on a small sample, all cases that developed Clavien-Dindo Grade IIIb complications following surgical repair of Type III or Type IV esophageal hiatal hernias were observationally associated with a large hernia size and prolonged operative time. Furthermore, the areas of the hernia sac measured on preoperative CT scans were significantly associated with operative time. Given the limited sample size, we used a parsimonious Firth’s penalized logistic regression to mitigate small-sample bias; in this model, hernia sac area remained associated with prolonged operative time. These findings suggest that a large hernia size directly increases surgical complexity, which in turn may increase the risk of postoperative complications.

All three Grade IIIb cases in this study were classified as Type IV and exhibited markedly large hernia sacs. In large hiatal hernias, the operative field is often limited, and extensive adhesions between the intrasac organs can make dissection more technically demanding. Such intraoperative challenges not only prolong the surgical time but may also contribute to complications such as bleeding, perforation, or infection.

Similar findings have been reported in abdominal wall hernias, where a large hernial sac and reduced intra-abdominal domain were associated with higher risk of postoperative complications and recurrence rates [14,15]. Although studies that directly investigate the relationship between hiatal hernia size and postoperative complications are limited, several reports have demonstrated that larger hernias are associated with a higher risk of recurrence. For instance, Jones et al. reported that a hernia sac diameter > 5.0 cm was significantly associated with an increased risk of recurrence in a large patient population (n = 209) [16]. Grubnik and Malynovskyy found that recurrence occurred in 11.9% of patients (n = 598) with large hernias compared with 3.5% of those with small hernias [8]. Similarly, Armijo et al. showed that large hernias (>5.0 cm) were significantly more frequent in the recurrence group than in the non-recurrence group (82.9% vs. 54.2%, p = 0.006; n = 322) [17]. These data underscore the importance of quantifying hernia size during the preoperative evaluation. In addition to hernia size, prior studies have identified patient-related factors such as obesity, smoking, and diabetes mellitus as relevant to recurrence and postoperative morbidity; for example, Lidor et al. found that BMI and smoking status were considered in their analysis of paraesophageal hernia repair outcomes [18]. El-Magd et al. demonstrated that Type IV hernias (larger defects) were associated with increased perioperative complications, underscoring the interplay between anatomical and patient factors [19].

In the present study, the three cases that developed Grade IIIb complications involved either early recurrence or esophageal perforation. All patients required a prolonged operative time because of intra-sac adhesions. Although vagus nerve preservation was attempted in the recurrent cases, this may have contributed to technical challenges during repair. It is presumed that the combination of a pre-existing short esophagus, strong traction during surgery, and sutures placed for esophageal fixation contributed to the injury.

Reflecting on these cases, preventive strategies such as gastropexy should be considered for patients with large hernias who are at a higher risk of recurrence and complications [20-22]. Although vagus nerve preservation is generally preferred to maintain gastric function, selective vagotomy may be justified in cases where tension release or detorsion is necessary to achieve secure repair.

This study had several limitations that should be acknowledged. Intraoperative factors could not be analyzed because operative videos were not available for all cases, and no standardized intraoperative documentation was recorded. This was a retrospective, single-center study with a relatively small sample size (n = 34). Notably, only three patients developed Clavien-Dindo Grade IIIb complications requiring reoperation, which limited the statistical power of the findings. Accordingly, the multivariate analysis was likely underpowered, and the results should be interpreted as exploratory rather than confirmatory. In addition, the absence of an external validation cohort limits the generalizability of the findings. Future multicenter prospective studies with larger cohorts are warranted to better elucidate the effects of hernia size and intraoperative factors on postoperative outcomes.

## Conclusions

This study showed that in all cases resulting in Clavien-Dindo Grade IIIb complications, patients presented with large hiatal hernias and required prolonged operative times. A large hernia size may increase the technical difficulty of surgery and thus represents a potential risk factor for serious postoperative complications. Thus, accurate preoperative assessment of the hernia size is essential for appropriate surgical planning and intraoperative strategies, which may help minimize postoperative risks. In clinical practice, incorporating quantitative CT measurements such as hernia sac area into preoperative assessment could facilitate operative scheduling, allocation of experienced surgeons, and informed patient counseling, ultimately contributing to safer surgical management. For early-career surgeons, it is particularly important to anticipate preoperative factors that may prolong operative time and to plan early involvement of senior surgeons when appropriate, as well as to perform meticulous adhesiolysis and careful suturing around the hiatus to minimize the risk of complications.
